# The Pharmaceutists’ and Druggists’ Practical Receipt-Book, with a Glossary of Medical Terms, and Copious Index

**Published:** 1865-04

**Authors:** 


For sale by S. C. Griggs & Co., 41 Lake Street, Chicago.

---

The Pharmaceutists' and Druggists' Practical Receipt-Book, with a Glossary of Medical Terms, and Copious Index. By Thomas F. Branston. Philadelphia: Lindsay & Blakiston. 1865.

This is a duodecimo volume of 307 pages. Its contents are well indicated by its title: it being made up of recipes, formulas, explanations of terms, &c. The subjects are conveniently arranged in alphabetical order, and the book is altogether a convenient one for reference, both by physicians and druggists.

